# Association of NCAP family genes with prognosis and immune infiltration of human sarcoma

**DOI:** 10.18632/aging.204683

**Published:** 2023-05-09

**Authors:** Guangyao Jiang, Qunyan Tian, Peikai Shi, Zhigao Li, Yan Li, Junjie Chen, Wanchun Wang, Ruiqi Chen, Hua Zhong, Gen Wu

**Affiliations:** 1 Department of Orthopedics, People’s Hospital of Pingchang County, Pingchang, Sichuan 636400, China; 2 Department of Orthopedics, The Second Xiangya Hospital of Central South University, Changsha, Hunan 410011, China; 3 The Fourth Clinical Medical College of Guangzhou University of Chinese Medicine, Shenzhen, Guangdong 518000, China; 4 Department of General Surgery, People’s Hospital of Pingchang County, Pingchang, Sichuan 636400, China; 5 Department of Orthopedics, Longhui People’s Hospital, Shaoyang, Hunan 422200, China; 6 Department of Orthopedics, The Fifth Affiliated Hospital, Southern Medical University, Guangzhou, Guangdong 510900, China

**Keywords:** expression, NCAP family genes, prognosis, immune infiltration, sarcoma

## Abstract

Objective: This study was conducted to explore the correlation of NCAP family genes with expression, prognosis, and immune infiltration in human sarcoma.

Results: Compared with normal human tissues, six NCAP family genes were highly expressed in sarcoma tissues, and high expression of the six genes were significantly associated with the poor prognosis of sarcoma patients. The expression of NCAPs in sarcoma was significantly related to the low infiltration level of macrophages and CD4+ T cells. GO and KEGG enrichment analysis showed that NCAPs and their interacting genes were mainly enriched in organelle fission for biological processes (BP), spindle for cellular component (CC), tubulin binding for molecular function (MF), and ‘Cell cycle’ pathway.

Methods: We explored the expression of NCAP family members by ONCOMINE, and GEPIA databases. Additionally, the prognostic value of NCAP family genes in sarcoma was detected by Kaplan-Meier Plotter and GEPIA databases. Moreover, we explored the relationship between NCAP family gene expression level and immune infiltration using the TIMER database. Finally, we performed GO and KEGG analysis for NCAPs-related genes by DAVID database.

Conclusion: The six members of NCAP gene family can be used as biomarkers to predict the prognosis of sarcoma. They were also correlated with the low immune infiltration in sarcoma.

## INTRODUCTION

Sarcoma is a set of mesenchymal and heterogeneous neoplasms, which principally is grouped into primary osteosarcoma and soft tissue sarcoma and contains beyond 100 different diagnostic entities [1]. Several causative factors of sarcoma have been identified, including Germline mutations, radiation, and carcinogens [2]. Osteosarcoma in adolescents is the most frequent occurrence of primary malignant bone tumor [3] and has an undesirable mortality rate. [4]. The 5-year survival rate is relatively low, about 60%–70% [5], and the 5-year survival rate of patients with metastatic osteosarcoma is only 19–30% [6]. Even with the progress of surgical and auxiliary treatment, the molecular mechanism of sarcoma needs to be further explored [7, 8].

Condensins participate in cell mitosis and meiosis in the human cell cycle [9]. Condensins are involved in chromosome aggregation and separation in the cell cycle [10]. There are two kinds of condensins in human cells, condensin I and condensin II [11]. Condensin I complex comprises three non-SMC subunits and structural maintenance of chromosomes (SMC) proteins [12]. Three non-SMC subunits are named non-SMC condensin I complex H subunit (NCAPH), non-SMC condensin I complex G subunit (NCAPG), and non-SMC condensin I complex D2 subunit (NCAPD2) separately [13]. Condensin II complex combines with enhancer and promoter during DNA transcription and can regulate the expression of genes [14, 15]. Non-SMC condensin I complex H subunit (NCAPH), non-SMC condensin I complex G subunit (NCAPG2) and non-SMC condensin I complex D2 subunit (NCAPD3) are subunits of condensins II [16, 17].

**Figure 1:**
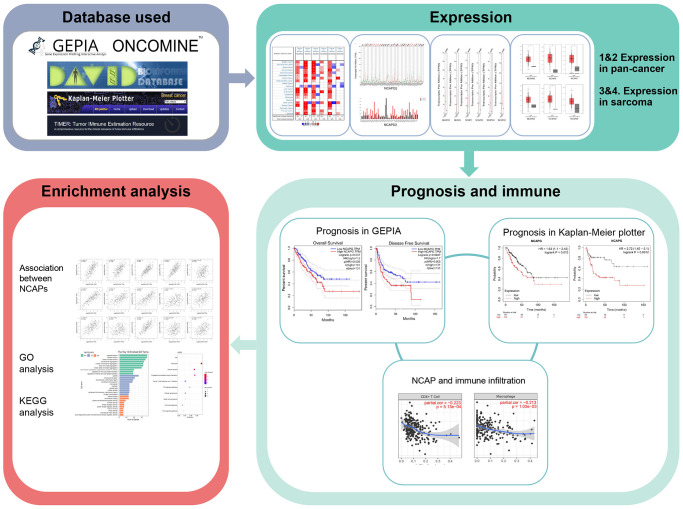
Flow chart of the study.

Up to now, NCAPD2, NCAPG, NCAPH, NCAPD3, NCAPG2, and NCAPH2 have not been mentioned in sarcoma, except NCAPG mentioned in Ewing sarcoma [18]. In this study, ONCOMINE, GEPIA, Kaplan-Meier plotter, DAVID (KEGG and GO analysis), and TIMER datasets were utilized to study the high expression, prognosis analysis, signal pathway and immune correlation of NCAPD2, NCAPG, NCAPH, NCAPD3, NCAPG2, and NCAPH2 in sarcoma for the first time (Figure 1). NCAP family genes are expected to become early prognostic biomarkers of sarcoma patients.

## RESULTS

### Transcriptional levels of NCAPs in sarcoma patients

**Figure 2:**
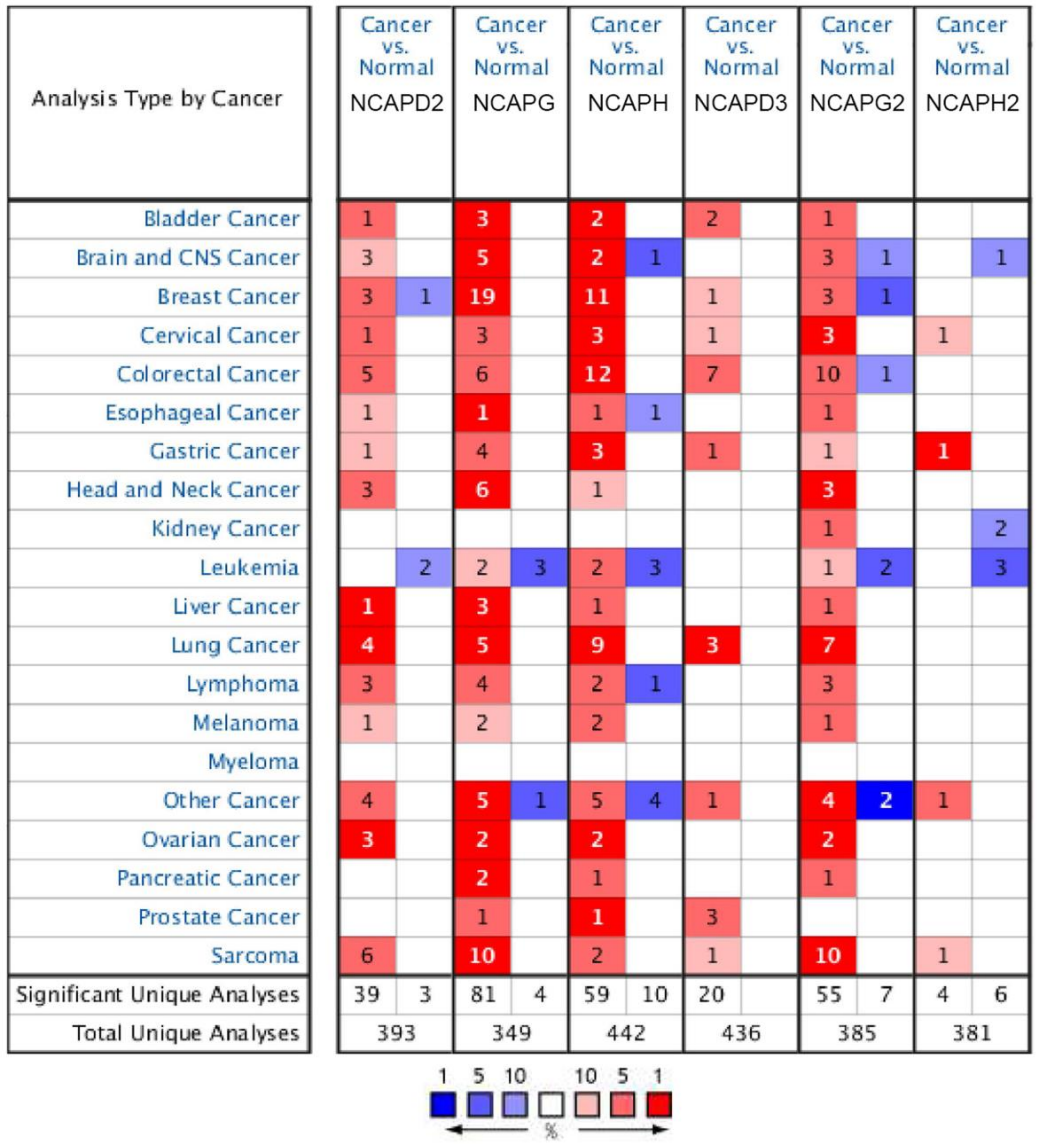
**The expression levels of NCAP genes in different types of human cancers and normal samples.** The red cells represent evidence of gene overexpression. The blue cells represent evidence of reduced gene expression. The numbers in each cell represent the evidential frequencies. The deeper the color, the higher the significance.

In our research, differential expression levels of NCAPs between normal tissues and cancer tissues were analyzed by ONCOMINE and GEPIA databases. Six members of the NCAPs family, including NCAPD2, NCAPG, NCAPH, NCAPD3, NCAPG2, and NCAPH2, have been found in different types of sarcomas. All of them were upregulated in sarcoma samples compared to normal tissues in 2 databases (Figure 2).

Firstly, in Detwiller Sarcoma dataset, NCAPD2 was overexpressed in Maligant Fibrous Histiocytoma, Fibrosarcoma and Leiomyosarcoma with 2.866 (*p* = 4.03E-6), 2.661 (*p* = 1.25E-4) and 2.690 (*p* = 0.002) fold changes. NCAPG was upregulated in Leiomyosarcoma, Pleomorphic Liposarcoma, Fibrosarcoma, Malignant Fibrous Histiocytoma, Round Cell Liposarcoma and Synovial Sarcoma. The fold changes of these sarcomas were as follows: 41.047 (*p* = 4.10E-10), 53.462 (*p* = 4.87E-10), 40.123 (*p* = 7.77E-10), 47.334 (*p* = 2.31E-10), 27.533 (5.79E-9) and 18.076 (8.4E-7). NCAPH was upregulated in Malignant Fibrous Histiocytoma and Fibrosarcoma with fold changes of 11.886 (*p* = 5.22E-6) and 10.772 (*p* = 7.72E-5). NCAPD3 was upregulated in Round Cell Liposarcoma with a fold change of 2.213 (*p* = 0.003). NCAPG2 was upregulated in Pleomorphic Liposarcoma, Leiomyosarcoma, Malignant Fibrous Histiocytoma, Fibrosarcoma, Dedifferentiated Liposarcoma, Synovial Sarcoma, and Round Cell Liposarcoma. The fold changes were as follows: 11.472 (*p* = 3.13E-6), 9.095 (*p* = 1.28E-6), 8.265 (*p* = 2.05E-6), 6.340 (*p* = 1.30E-5), 3.671 (*p* = 0.002), 5.076 (*p* = 5.20E-5) and 5.645 (*p* = 2.72E-4). NCAPH2 was upregulated in Round Cell Liposarcoma with a fold change of 2.013 (*p* = 0.002). In the Barretina Sarcoma dataset, NCAPD2 was overexpressed in Myxofibrosarcoma, Pleomorphic Liposarcoma, and Leiomyosarcoma with 2.627 (*p* = 4.43E-9), 2.118 (*p* = 1.21E-6) and 2.361 (*p* = 9.55E-8) fold changes. NCAPG was upregulated in Myxofibrosarcoma, Pleomorphic Liposarcoma, and Leiomyosarcoma with 3.118 (*p* = 4.55E-14), 3.051 (*p* = 7.64E-10) and 2.633 (*P* = 6.60E-8) fold changes. NCAPG2 was upregulated in Pleomorphic Liposarcoma, Leiomyosarcoma, and Myxofibrosarcoma with 2.388 (*p* = 1.98E-8), 2.540 (*p* = 1.69E-9) and 2.175 (*p* = 1.36E-9) fold changes (Table 1).

**Table 1:** The significant changes of NCAPs expression in transcription level between different types of sarcoma.

**Gene ID**	**Types of sarcoma vs. Normal**	**Fold change**	***p* Value**	***t* Test**	**References**
NCAPD2	Malignant Fibrous Histiocytoma vs. Normal	2.866	4.03E-6	6.926	Detwiller Sarcoma
	Fibrosarcoma vs. Normal	2.661	1.25E-4	5.742	Detwiller Sarcoma
	Leiomyosarcoma vs. Normal	2.690	0.002	4.291	Detwiller Sarcoma
	Myxofibrosarcoma vs. Normal	2.627	4.43E-9	8.927	Barretina Sarcoma
	Pleomorphic Liposarcoma vs. Normal	2.118	1.21E-6	5.938	Barretina Sarcoma
	Leiomyosarcoma vs. Normal	2.361	9.55E-8	6.805	Barretina Sarcoma
NCAPG	Leiomyosarcoma vs. Normal	41.047	4.10E-10	11.716	Detwiller Sarcoma
	Pleomorphic Liposarcoma vs. Normal	53.462	4.87E-10	13.338	Detwiller Sarcoma
	Fibrosarcoma vs. Normal	40.123	7.77E-10	10.439	Detwiller Sarcoma
	Malignant Fibrous Histiocytoma vs. Normal	47.334	2.31E-10	10.536	Detwiller Sarcoma
	Round Cell Liposarcoma vs. Normal	27.533	5.79E-9	10.209	Detwiller Sarcoma
	Synovial Sarcoma vs. Normal	18.076	8.40E-7	7.926	Detwiller Sarcoma
	Myxofibrosarcoma vs. Normal	3.118	4.55E-14	11.534	Barretina Sarcoma
	Pleomorphic Liposarcoma vs. Normal	3.051	7.64E-10	9.116	Barretina Sarcoma
	Leiomyosarcoma vs. Normal	2.633	6.60E-8	7.124	Barretina Sarcoma
NCAPH	Malignant Fibrous Histiocytoma vs. Normal	11.886	5.22E-6	7.869	Detwiller Sarcoma
	Fibrosarcoma vs. Normal	10.722	7.72E-5	6.961	Detwiller Sarcoma
NCAPD3	Round Cell Liposarcoma vs. Normal	2.213	0.003	3.265	Detwiller Sarcoma
NCAPG2	Pleomorphic Liposarcoma vs. Normal	11.472	3.13E-6	7.015	Detwiller Sarcoma
	Leiomyosarcoma vs. Normal	9.095	1.28E-6	7.015	Detwiller Sarcoma
	Malignant Fibrous Histiocytoma vs. Normal	8.265	2.05E-6	6.320	Detwiller Sarcoma
	Fibrosarcoma vs. Normal	6.340	1.30E-5	5.504	Detwiller Sarcoma
	Dedifferentiated Liposarcoma vs. Normal	3.671	0.002	3.419	Detwiller Sarcoma
	Synovial Sarcoma vs. Normal	5.076	5.20E-5	5.050	Detwiller Sarcoma
	Round Cell Liposarcoma vs. Normal	5.645	2.72E-4	4.529	Detwiller Sarcoma
	Pleomorphic Liposarcoma vs. Normal	2.388	1.98E-8	7.478	Barretina Sarcoma
	Leiomyosarcoma vs. Normal	2.540	1.69E-9	8.105	Barretina Sarcoma
	Myxofibrosarcoma vs. Normal	2.175	1.36E-9	7.718	Barretina Sarcoma
NCAPH2	Round Cell Liposarcoma vs. Normal	2.013	0.002	3.252	Detwiller Sarcoma

**Figure 3:**
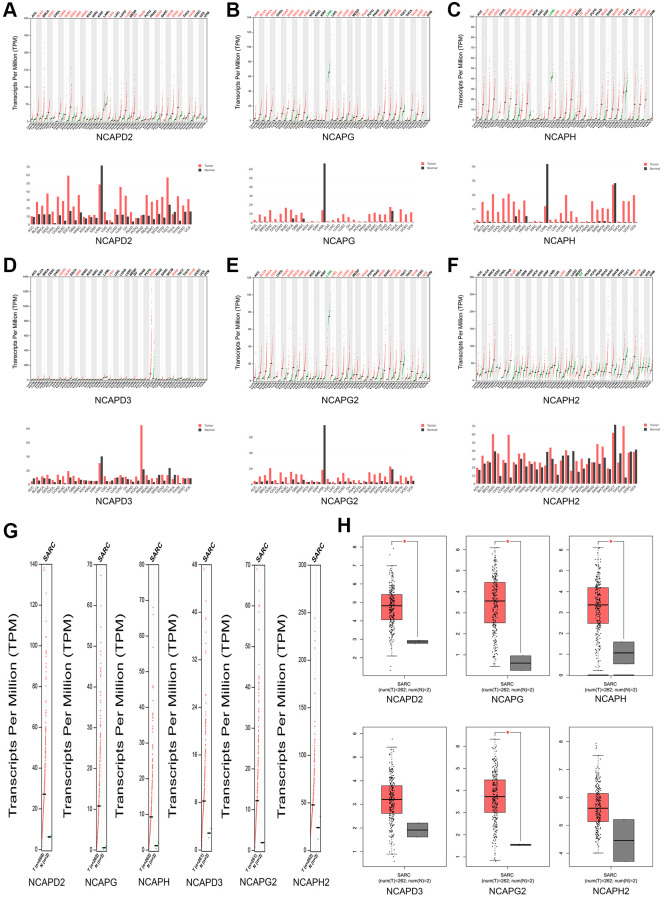
**The expression levels of NCAP genes in sarcoma.** (**A**–**F**) The expression levels of NCAPD2, NCAPG, NCAPD3, NCAPG2, NCAPH, and NCAPH2 in pan-cancer, (**G**, **H**) The expression levels of NCAP genes in sarcoma. Each dot represents an individual sample, ^*^P < 0.05.

By evaluating NCAPs in normal and sarcoma tissues using the GEPIA database, we noticed significantly higher expression patterns of NCAPD2, NCAPG, NCAPH, NCAPG2, and in sarcoma samples compared to normal tissues. NCAPD3 and NCAPH2 were also upregulated in sarcoma samples but with no significance (Figure 3).

### The prognostic value of high expression of NCAPs in sarcoma

**Figure 4:**
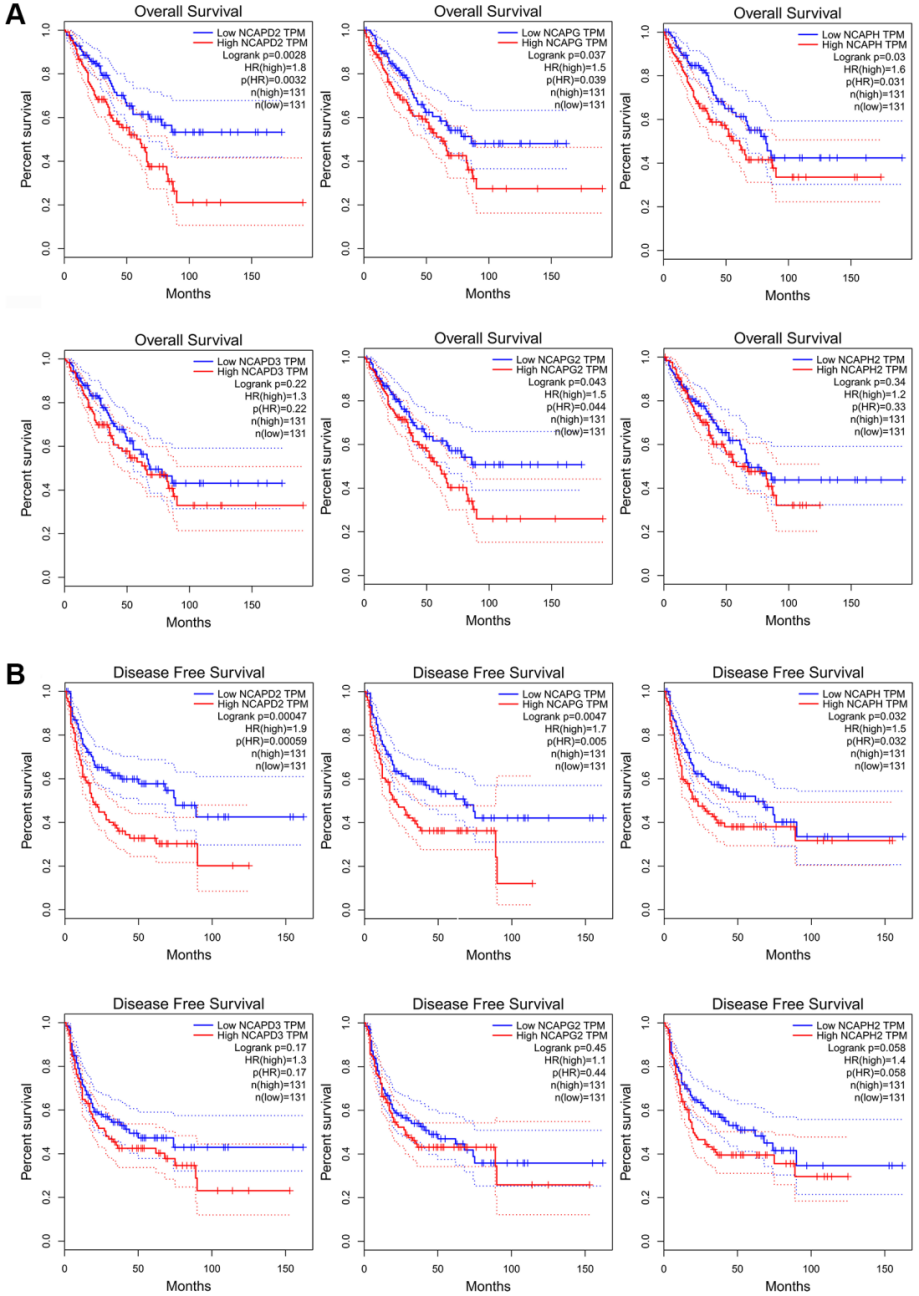
**The prognostic value of mRNA level of NCAP factors in sarcoma patients.** Higher expression of NCAP genes is associated with worse (**A**) overall survival and (**B**) disease-free survival. Abbreviation: HR: hazard ratio.

We investigated the clinical prognostic value of NCAPs in sarcoma using the Kaplan-Meier Plotter and GEPIA databases and obtained survival data for patients with sarcoma. GEPIA database showed that high expression of NCAPD2, NCAPH, NCAPG and NCAPG2 had a significant correlation with poor overall survival of sarcoma patients (*P* < 0.05). The high expression of NCAPD3 and NCAPH2 were also associated with poor overall survival but there was no statistical significance (*P* > 0.05). The overall survival hazard ratios of NCAPD2, NCAPG, NCAPH, and NCAPG2 were 1.8 (*P* = 0.0028), 1.5 (*P* = 0.037), 1.6 (*P* = 0.03) and 1.5 (*P* = 0.043) respectively (Figure 4A). The Figure 4B showed that high expression levels of NCAPD2, NCAPG, and NCAPH were significantly correlated with the poor disease free survival of sarcoma patients. The disease free survival hazard ratios of NCAPD2, NCAPG, and NCAPH were 1.9 (*P* = 0.00047), 1.7 (*P* = 0.0047), and 1.5 (*P* = 0.032), respectively.

**Figure 5:**
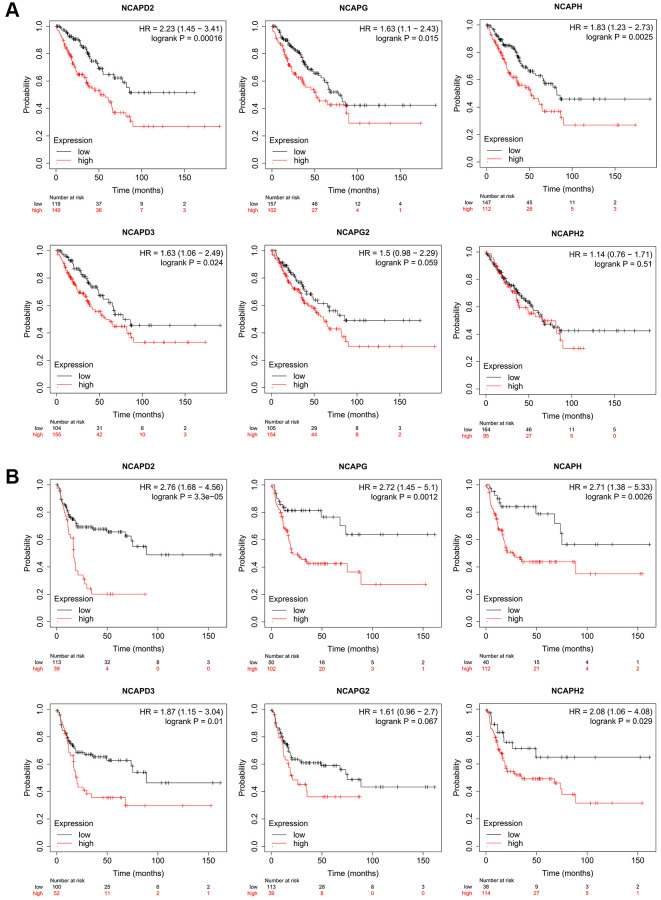
**The prognostic value of mRNA level of NCAP factors in sarcoma patients.** The prognostic value of mRNA level of NCAP factors in sarcoma patients was analyzed by Kaplan Meier Plotter. Expression of NCAP family genes was related to poor (**A**) overall survival and (**B**) relapse-free survival in sarcoma.

In the Kaplan-Meier Plotter database, high expression of NCAPD2, NCAPG, NCAPH, and NCAPD3 was correlated with poor overall survival in sarcoma patients (*p* < 0.05), HR = 2.23 (*p* = 0.00016), 1.63 (*p* = 0.015), 1.83 (*p* = 0.0025), and 1.63 (*p* = 0.024), respectively (Figure 5A). The high expression of NCAPD2, NCAPG, NCAPH, NCAPD3, and NCAPH2 were all correlated with the relapse-free survival of sarcoma patients, HR = 2.76 (*p* = 3.3e-05), 2.72 (*p* = 0.0012), 2.71 (*p* = 0.0026), 1.87 (*p* = 0.01) and 2.08 (*p* = 0.029) respectively (Figure 5B). In addition, the high expression of NCAPG2 was also correlated with the poor overall survival and disease-free survival of sarcoma patients, but with no significance (*p* > 0.05). The high expression of NCAPH2 was correlated with poor overall survival of sarcoma patients but had no statistical significance (*p* > 0.05).

### Association between NCAP genes

**Figure 6:**
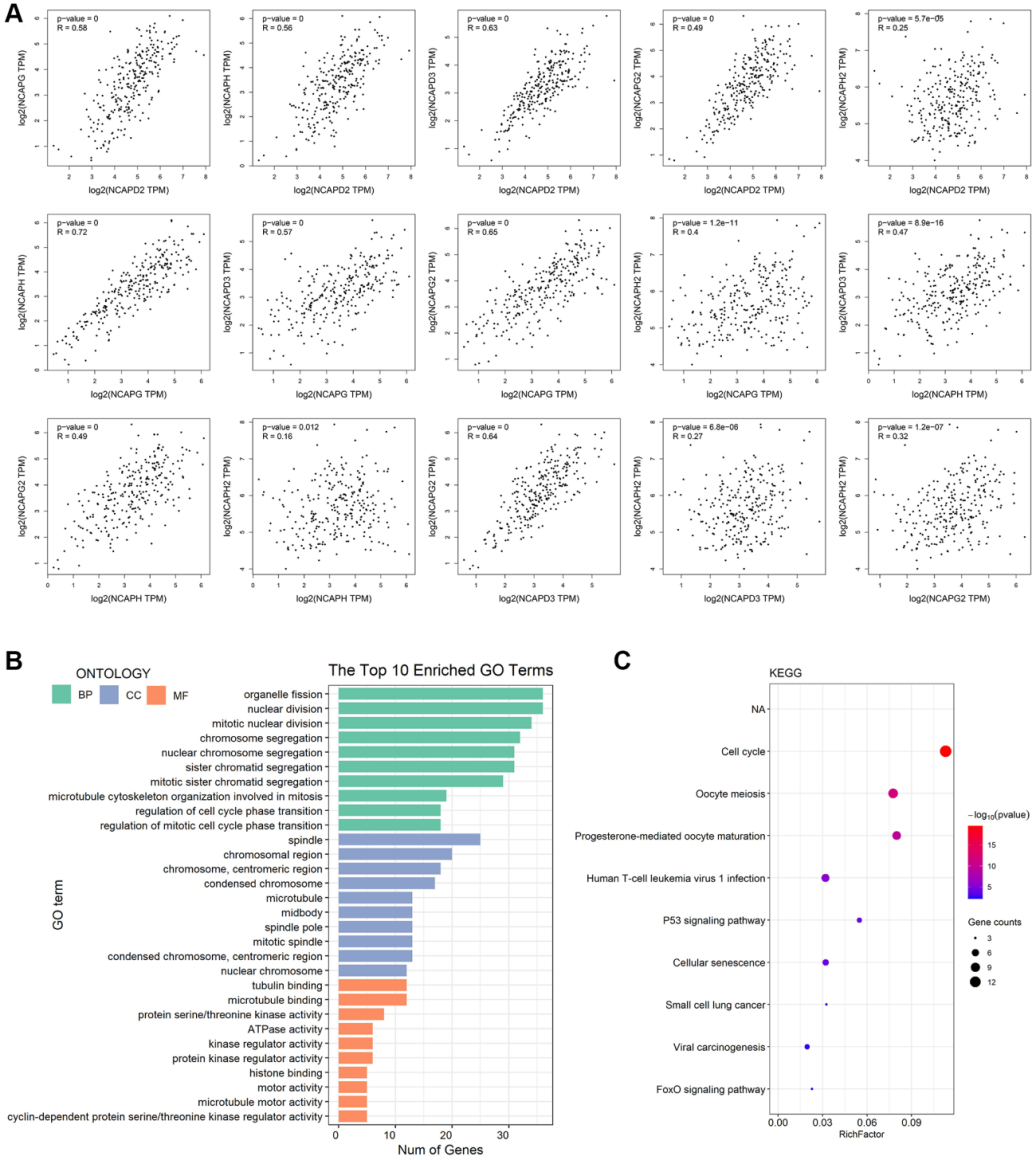
**Co-expressed analysis, GO analysis, and functional enrichment analysis of NCAP and their interacting genes.** (**A**) Correlation between NCAP genes in sarcoma. (**B**) GO analysis was based on three aspects. Abbreviations: BP: biological processes; CC: cellular components; MF: molecular function. (**C**) KEGG pathway related to NCAP genes and their interacting genes in sarcoma.

The present study investigated the association between the expression of NCAPD2, NCAPG, NCAPH, NCAPD3, NCAPG2, and NCAPH2 by using the GEPIA database. The study found that there were positive correlations between NCAPD2 and NCAPG (R = 0.58 *p* < 0.05), NCAPH and NCAPD2 (R = 0.56 *p* < 0.05), NCAPD3 and NCAPD2 (R = 0.63 *p* < 0.05), NCAPG2 and NCACPD2 (R = 0.49 *p* < 0.05), NCAPH and NCAPG (R = 0.72 *p* < 0.05), NCAPD3 and NCAPG (R = 0.57 *p* < 0.05), NCAPG2 and NCAPG (R = 0.65 *p* < 0.05), NCAPH2 and NCAPG (R = 0.4 *p* < 0.05), NCAPD3 and NCAPH (R = 0.47 *p* < 0.05), NCAPG2 and NCAPH (R = 0.49 *p* < 0.05), NCAPG2 and NCAPD3 (R = 0.64 *p* < 0.05), NCAPH2 and NCAPG2 (R = 0.32 *p* < 0.05). But there was no linear correlation between NCAPH2 and NCAPD2, NCAPH2 and NCAPH, NCAPH2 and NCAPH (R < 0.3) (Figure 6A).

### Predicted functions and pathways of NCAPs and their 50 interacting genes in sarcoma

We performed GO function enrichment analysis and KEGG pathway analysis of NCAPs and their 50 interacting genes using DAVID database. The results showed that these genes were mainly involved in the biological processes (BPs) of organelle fission, nuclear division and mitotic nuclear division, cellular components (CCs) of spindle, chromosomal region, chromosome centromeric region, and molecular functions (MFs) of tubulin binding, microtubule binding and protein serine/threonine kinase activity (Figure 6B). In addition, KEGG pathway analysis indicated that NCAPs and their 50 interacting genes played a vital role in the cell cycle, oocyte meiosis, and progesterone-mediated oocyte maturation (Figure 6C).

### The relationship between the expression level of the NCAPs family and immune cell infiltration

**Figure 7:**
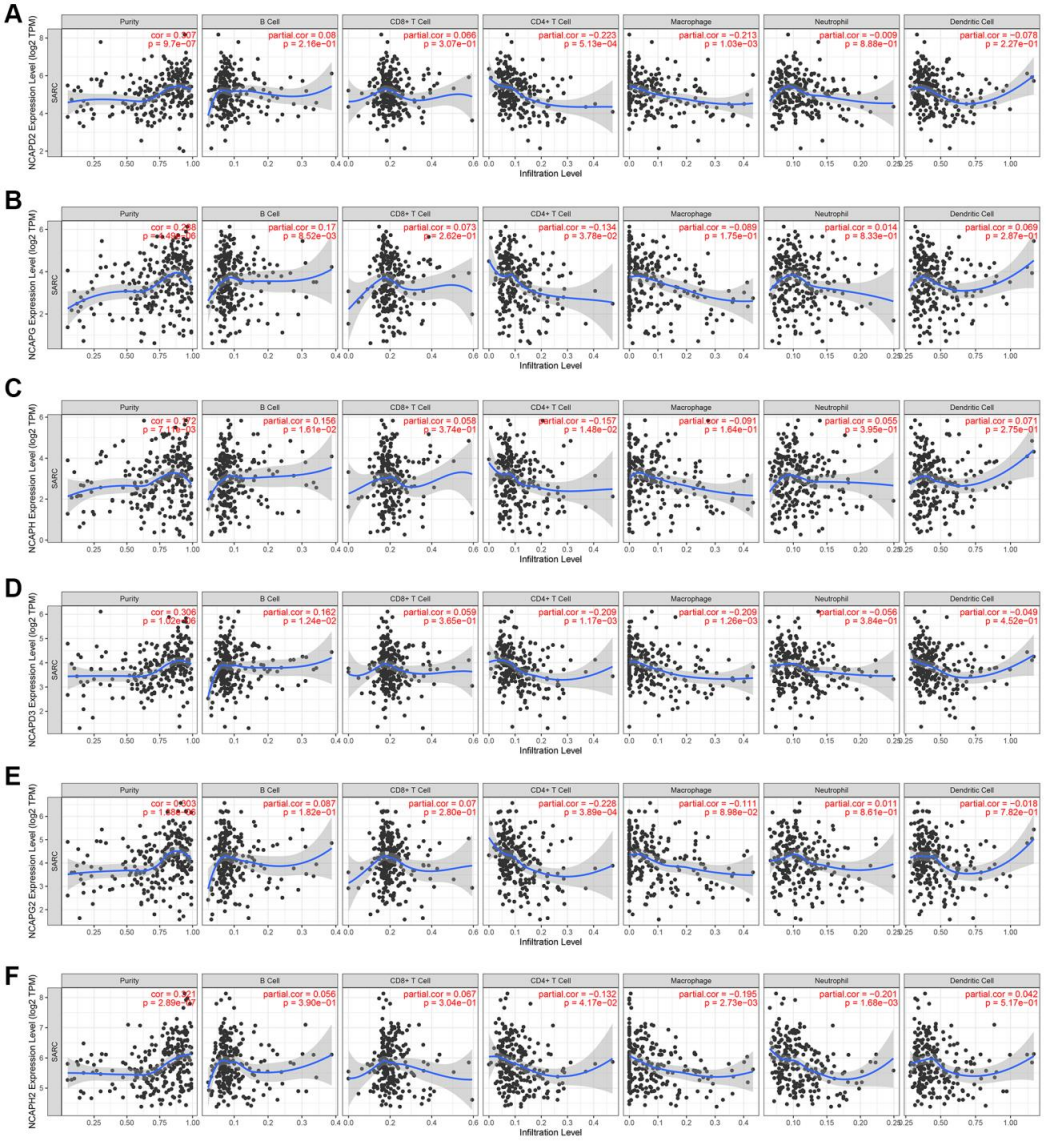
**Analysis of immune infiltration.** Relationship of differentially expressed (**A**–**F**) NCAPD2, NCAPG, NCAPH, NCAPD3, NCAPG2, and NCAPH2 with immune cell infiltration. The immune cells we analyzed included B cells, CD8+ T cells, CD4+ T cells, macrophages, neutrophils, and dendritic cells.

Using the TIMER database, we analyzed the relationship between NCAP gene family and immune cell infiltration (Figure 7). The results showed that high expression of NCAPD2 was significantly associated with low infiltration levels of CD4+ T cells (correlation coefficient (cor) = −0.223, *p* < 0.05) and macrophages (cor = −0.213, *p* < 0.05). Similarly, the expression levels of NCAPG (cor = −0.134, *p* < 0.05), NCAPH (cor = −0.157, *p* < 0.05), NCAPD 3 (cor = −0.209, *p* < 0.05), NCAPG 2 (cor = −0.228, *p* < 0.05), and NCAPH 2 (cor = −0.132, *p* < 0.05) were significantly negatively correlated with the level of CD4+ T cell infiltration. The expression levels of NCAPD2 (cor = −0.213, *p* < 0.05), NCAPD3 (cor = −0.209, *p* < 0.05) and NCAPH2 (cor = −0.195, *p* < 0.05) were significantly negatively correlated with the macrophage infiltration level.

## DISCUSSION

Sarcoma is a group of heterogeneous interstitial tumors with more than 100 different diagnostic entities. It is characterized by high malignancy, poor prognosis, and low survival rate. Therefore, it is crucial to find biomarkers for the early prediction of sarcoma. In our study, this is the first time to explore the possibility of using NCAP family genes as biomarkers for sarcoma prognosis.

Bioinformatics analysis is a subject that studies biology by using informatics, applied mathematics, computer science, and statistics [19]. In this study, the expression of NCAP members in sarcoma, signal pathway in sarcoma, immune cell infiltration in sarcoma microenvironment, and its influence on the prognosis of sarcoma patients were studied by bioinformatics analysis. In addition, we explored the correlation between all NCAP family genes. Through the study of the ONCOMINE and GEPIA databases, we found that four members of the NCAP gene family were highly expressed in sarcoma compared with normal human tissue. In addition, the expression of NCAP family genes was explored in this study and we found that NCAP family genes were positively correlated with each other. What’s more, we also confirmed that the high expression of NCAP family members was significantly related to the immune infiltration level of CD4+ T cells and macrophages, which provided a new insight for the effectiveness of future immunotherapy for sarcoma.

Previously, studies have found that NCAPD2 could promote breast cancer progression [20]. NCAPD2 plays a role in colorectal cancer through Ca2+/CAMKK/AMPK/mTORC1 pathway and PARP-1/SIRT1 axis and can be used as a potential therapeutic target [21]. Our research confirmed the value of high expression of NCAPD2 in the prognosis of human sarcoma by using the Kaplan-Meier plotter and GEPIA database, and found that it was negatively correlated with the OS and DFS of sarcoma patients.

Similarly, NCAPH can be used as a prognostic biomarker for non-small cell lung cancer, lung adenocarcinoma, nasopharyngeal carcinoma, breast cancer, and hepatocellular carcinoma [22–26]. In addition, Meng Wang et al. found that NCAPH regulated the occurrence of cervical cancer through the PI3K/AKT/SGK pathway [27]. With Kaplan-Meier Plotter, we verified the prognostic value of NCAPH in sarcoma: the high expression of NCAPH was negatively correlated with OS and RFS, and NCAPH can be used as a promising potential biomarker for sarcoma.

As for NCAPG, it has been proved to be involved in the development of prostate cancer, lung adenocarcinoma, hepatocellular carcinoma, and non-small cell lung cancer [28–31]. However, our research proved that the high expression of NCAPG in sarcoma is relevant to the lower OS and DFS of sarcoma patients.

High expression of NCAPG2 has been shown to be associated with poor prognosis in patients with lung adenocarcinoma and hepatocellular carcinoma [14, 32]. In addition, the research of Jianheng Wu et al. demonstrated that NCAPG2 in glioblastoma promoted cell malignant transformation and activation by phosphorylating HBO1 [33]. In GEPIA dataset, we verified that the overexpression of NCAPG2 was positively correlated with low DFS of sarcoma patients.

Abnormal methylation of NCAPH2 in the blood of patients with subjective cognitive decline may be a biomarker for early screening of Alzheimer’s disease [17], but no related research has mentioned that NCAPH2 was related to cancer prognosis. In our research, we also found that the overexpression of NCAPH2 had statistical significance in correlation with low RFS of sarcoma patients.

What’s more, high expression of NCAPD3 was confirmed to be related to the occurrence and progression of the tumor by Zuolei Jing et al. in chromatic cancer, and overexpression of NCAPD3 led to poor prognosis of chromatic cancer [34]. From our research results, we also found that high expression of NCAPD3 was related to the poor prognosis of sarcoma in the Kaplan-Meier Plotter database.

In addition, we used GO analysis to analyze the biological processes involved in the NCAPs gene family and their interacting genes. The results showed that NCAPs and their interacting genes were mainly involved in the biological processes and molecular functions related to the cell division and were mainly expressed in structures related to cell division. In addition, KEGG analysis also showed that NCAPs gene family and their interacting genes were mainly concentrated in cell cycle-related signaling pathways. These results suggested that the abnormal expression of NCAPs and its interacting genes may be involved in the occurrence and development of tumors by altering the normal cell cycle. The cell cycle is a complex process that involves many regulatory proteins everywhere in organisms [35]. Previously, PJan M Suski et al. showed that abnormal activity of core cell cycle mechanism existed in different types of tumors, so inhibition the expression of different cell cycle proteins can be used as a new strategy for tumor treatment [36].

In our study, we explored the expression of NCAPs in sarcoma, which had important value for the prognosis of sarcoma patients. In other words, the high expression of these genes often leads to a more malignant sarcoma. Therefore, the six members of the NCAP family genes are all expected to become an important biomarker for sarcoma diagnosis and prognosis prediction and the expression level of NCAPs gene family were all related to the infiltration level of immune cells in sarcoma tumor microenvironment. However, our research still had some limitations. First of all, we used all the existing data but needed more experimental verification, including histological analysis and qPCR. Secondly, this study only explored the NCAP family genes in sarcoma tissues but did not study the expression of NCAPs in the blood of sarcoma patients. If we can further research the expression of NCAPs in the peripheral blood of sarcoma patients, it is expected to become a more convenient diagnostic screening method.

## CONCLUSIONS

In a word, we confirmed the high expression of the NCAPs family in sarcoma by bioinformatics analysis, the high expression of the six members of NCAP gene family were all connected with the prognosis and immune infiltration of sarcoma, and their high expression provided a novel target for the therapy of sarcoma and a new insight for the efficacy of immunotherapy of sarcoma.

## METHODS

### ONCOMINE (http://www.oncomine.org/) analysis

The ONCOMINE database was usually utilized to investigate the NCAP family’s differential expression and clinical relevance in various cancers. The expression information of the NCAP family in sarcoma and the clinical correlation of NCAPs genes were retrieved from ONCOMINE, with default settings of fold change > 2 and *P* value < 0.05. The ONCOMINE database was discontinued in 2022.

### GEPIA (http://gepia.cancer-pku.cn/) analysis

Gene Expression Profiling Interactive Analysis (GEPIA) is a web server based on normal gene and cancer expression profiling and interactive analysis [37]. In this study, GEPIA was extensively applied to investigate the distinct expression and the prognostic value of the NCAP family genes in sarcoma and correlation analysis between NCAP family members, with default setting *p* < 0.05.

### Kaplan-Meier plotter (http://kmplot.com/analysis/) analysis

In this study, the Kaplan-Meier plotter was used to explore the connection between the prognosis of sarcoma patients and different expressions of NCAPs, including relapse free survival and overall survival. Kaplan Meier Plotter contains survival information for 21 diverse types of cancer, containing sarcomas, and expression data for 54,000 genes. In addition, Kaplan-Meier plotter compares NCAPs mRNA high and low expression levels in samples to correlate prognosis with hazard ratios (HR) and log-rank *P* values with 95% confidence intervals.

### KEGG and GO enrichment analyses of NACPs and their interacting genes

Kyoto Encyclopedia of Genes and Genomes (KEGG) pathway and Gene Ontology (GO) Enrichment analysis of NCAP gene family and their interacting genes were performed using the DAVID database. DAVID (Database for Annotation, Visualization and Integrated Discovery) is an online tool and database based on bioinformatics that aims to assist researchers in annotating and analyzing large amounts of gene and protein data. The DAVID database contains a wide range of biological information and functional annotations, including Gene Ontology, biological pathways, disease associations, and more.

### TIMER dataset (https://cistrome.shinyapps.io/timer/) analysis

Tumor Immune Estimation Resource (TIMER) database pre-computed the levels of infiltrating immune systems in about six subsets for 10,897 tumors from 32 cancer types and investigated molecular characterization of tumor-immune interactions [38]. So, we took full advantage of the TIMER database to explore the expression of NCAPs in sarcoma and the correlation between the expression level of the NCAPs family and the infiltration level of different immune cells.

### Availability of data and materials

All data generated or analysed during this study are included in this published article.

## References

[1] Skubitz KM, D'Adamo DR. Sarcoma. Mayo Clin Proc. 2007; 82:1409–32. 10.4065/82.11.1409 17976362

[2] Popovich JR, Kashyap S, Gasalberti DP, Cassaro S. Sarcoma. In: StatPearls. Treasure Island (FL): StatPearls Publishing; 2023. 30137818

[3] Zhao X, Wu Q, Gong X, Liu J, Ma Y. Osteosarcoma: a review of current and future therapeutic approaches. Biomed Eng Online. 2021; 20:24. 10.1186/s12938-021-00860-0 33653371 PMC7923306

[4] Mattiuzzi C, Lippi G. Current Cancer Epidemiology. J Epidemiol Glob Health. 2019; 9:217–22. 10.2991/jegh.k.191008.001 31854162 PMC7310786

[5] Broadhead ML, Clark JC, Myers DE, Dass CR, Choong PF. The molecular pathogenesis of osteosarcoma: a review. Sarcoma. 2011; 2011:959248. 10.1155/2011/959248 21559216 PMC3087974

[6] Bishop MW, Janeway KA, Gorlick R. Future directions in the treatment of osteosarcoma. Curr Opin Pediatr. 2016; 28:26–33. 10.1097/MOP.0000000000000298 26626558 PMC4761449

[7] Smrke A, Anderson PM, Gulia A, Gennatas S, Huang PH, Jones RL. Future Directions in the Treatment of Osteosarcoma. Cells. 2021; 10:172. 10.3390/cells10010172 33467756 PMC7829872

[8] Blay JY. Intensity of recent years in the investigation of soft tissue sarcoma. Future Oncol. 2017; 13:3–9. 10.2217/fon-2017-0119 28460535

[9] Hirano T. Condensins: universal organizers of chromosomes with diverse functions. Genes Dev. 2012; 26:1659–78. 10.1101/gad.194746.112 22855829 PMC3418584

[10] Hirano T, Kobayashi R, Hirano M. Condensins, chromosome condensation protein complexes containing XCAP-C, XCAP-E and a Xenopus homolog of the Drosophila Barren protein. Cell. 1997; 89:511–21. 10.1016/s0092-8674(00)80233-0 9160743

[11] Kimura K, Cuvier O, Hirano T. Chromosome condensation by a human condensin complex in Xenopus egg extracts. J Biol Chem. 2001; 276:5417–20. 10.1074/jbc.C000873200 11136719

[12] Seipold S, Priller FC, Goldsmith P, Harris WA, Baier H, Abdelilah-Seyfried S. Non-SMC condensin I complex proteins control chromosome segregation and survival of proliferating cells in the zebrafish neural retina. BMC Dev Biol. 2009; 9:40. 10.1186/1471-213X-9-40 19586528 PMC2727499

[13] Schleiffer A, Kaitna S, Maurer-Stroh S, Glotzer M, Nasmyth K, Eisenhaber F. Kleisins: a superfamily of bacterial and eukaryotic SMC protein partners. Mol Cell. 2003; 11:571–5. 10.1016/s1097-2765(03)00108-4 12667442

[14] Ono T, Losada A, Hirano M, Myers MP, Neuwald AF, Hirano T. Differential contributions of condensin I and condensin II to mitotic chromosome architecture in vertebrate cells. Cell. 2003; 115:109–21. 10.1016/s0092-8674(03)00724-4 14532007

[15] Dowen JM, Bilodeau S, Orlando DA, Hübner MR, Abraham BJ, Spector DL, Young RA. Multiple structural maintenance of chromosome complexes at transcriptional regulatory elements. Stem Cell Reports. 2013; 1:371–8. 10.1016/j.stemcr.2013.09.002 24286025 PMC3841252

[16] Liu W, Tanasa B, Tyurina OV, Zhou TY, Gassmann R, Liu WT, Ohgi KA, Benner C, Garcia-Bassets I, Aggarwal AK, Desai A, Dorrestein PC, Glass CK, Rosenfeld MG. PHF8 mediates histone H4 lysine 20 demethylation events involved in cell cycle progression. Nature. 2010; 466:508–12. 10.1038/nature09272 20622854 PMC3059551

[17] Martin CA, Murray JE, Carroll P, Leitch A, Mackenzie KJ, Halachev M, Fetit AE, Keith C, Bicknell LS, Fluteau A, Gautier P, Hall EA, Joss S, et al, and Deciphering Developmental Disorders Study. Mutations in genes encoding condensin complex proteins cause microcephaly through decatenation failure at mitosis. Genes Dev. 2016; 30:2158–72. 10.1101/gad.286351.116 27737959 PMC5088565

[18] Zhao R, Xiong C, Zhang C, Wang L, Liang H, Luo X. Construction of a Prognosis-Related Gene Signature by Weighted Gene Coexpression Network Analysis in Ewing Sarcoma. Comput Math Methods Med. 2022; 2022:8798624. 10.1155/2022/8798624 35126643 PMC8814720

[19] Pallen MJ. Microbial bioinformatics 2020. Microb Biotechnol. 2016; 9:681–6. 10.1111/1751-7915.12389 27471065 PMC4993188

[20] He J, Gao R, Yang J, Li F, Fu Y, Cui J, Liu X, Huang K, Guo Q, Zhou Z, Wei W. NCAPD2 promotes breast cancer progression through E2F1 transcriptional regulation of CDK1. Cancer Sci. 2023; 114:896–907. 10.1111/cas.15347 35348268 PMC9986070

[21] Jing Z, He X, Jia Z, Sa Y, Yang B, Liu P. NCAPD2 inhibits autophagy by regulating Ca^2+^/CAMKK2/AMPK/mTORC1 pathway and PARP-1/SIRT1 axis to promote colorectal cancer. Cancer Lett. 2021; 520:26–37. 10.1016/j.canlet.2021.06.029 34229059

[22] Zhou W, Hu J, Zhao J. Non-SMC condensin I complex subunit H (NCAPH), a regulator of cell cycle, predicts poor prognosis in lung adenocarcinoma patients: a study mainly based on TCGA and GEO database. Transl Cancer Res. 2020; 9:7572–87. 10.21037/tcr-20-2217 35117357 PMC8798647

[23] Xiong Q, Fan S, Duan L, Liu B, Jiang X, Chen X, Xiong C, Tao Q, Wang J, Zhang H, Chen C, Duan Y. NCAPH is negatively associated with Mcl-1 in non-small cell lung cancer. Mol Med Rep. 2020; 22:2916–24. 10.3892/mmr.2020.11359 32945371 PMC7453632

[24] Xu L, Jiang Y, Zheng J, Xie G, Li J, Shi L, Fan S. Aberrant expression of β-catenin and E-cadherin is correlated with poor prognosis of nasopharyngeal cancer. Hum Pathol. 2013; 44:1357–64. 10.1016/j.humpath.2012.10.025 23375645

[25] Cui F, Hu J, Xu Z, Tan J, Tang H. Overexpression of NCAPH is upregulated and predicts a poor prognosis in prostate cancer. Oncol Lett. 2019; 17:5768–76. 10.3892/ol.2019.10260 31186803 PMC6507296

[26] Lu H, Shi C, Wang S, Yang C, Wan X, Luo Y, Tian L, Li L. Identification of NCAPH as a biomarker for prognosis of breast cancer. Mol Biol Rep. 2020; 47:7831–42. 10.1007/s11033-020-05859-9 33009967

[27] Wang M, Qiao X, Cooper T, Pan W, Liu L, Hayball J, Lin J, Cui X, Zhou Y, Zhang S, Zou Y, Zhang R, Wang X. HPV E7-mediated NCAPH ectopic expression regulates the carcinogenesis of cervical carcinoma via PI3K/AKT/SGK pathway. Cell Death Dis. 2020; 11:1049. 10.1038/s41419-020-03244-9 33311486 PMC7732835

[28] Liu W, Liang B, Liu H, Huang Y, Yin X, Zhou F, Yu X, Feng Q, Li E, Zou Z, Wu L. Overexpression of non-SMC condensin I complex subunit G serves as a promising prognostic marker and therapeutic target for hepatocellular carcinoma. Int J Mol Med. 2017; 40:731–8. 10.3892/ijmm.2017.3079 28737823 PMC5547945

[29] Gong C, Ai J, Fan Y, Gao J, Liu W, Feng Q, Liao W, Wu L. NCAPG Promotes The Proliferation Of Hepatocellular Carcinoma Through PI3K/AKT Signaling. Onco Targets Ther. 2019; 12:8537–52. 10.2147/OTT.S217916 31802891 PMC6801502

[30] Wu Y, Lin Y, Pan J, Tu X, Xu Y, Li H, Chen Y. NCAPG promotes the progression of lung adenocarcinoma via the TGF-β signaling pathway. Cancer Cell Int. 2021; 21:443. 10.1186/s12935-021-02138-w 34419073 PMC8380402

[31] Sun H, Zhang H, Yan Y, Li Y, Che G, Zhou C, Nicot C, Ma H. NCAPG promotes the oncogenesis and progression of non-small cell lung cancer cells through upregulating LGALS1 expression. Mol Cancer. 2022; 21:55. 10.1186/s12943-022-01533-9 35180865 PMC8855584

[32] Leiserson MD, Vandin F, Wu HT, Dobson JR, Eldridge JV, Thomas JL, Papoutsaki A, Kim Y, Niu B, McLellan M, Lawrence MS, Gonzalez-Perez A, Tamborero D, et al. Pan-cancer network analysis identifies combinations of rare somatic mutations across pathways and protein complexes. Nat Genet. 2015; 47:106–14. 10.1038/ng.3168 25501392 PMC4444046

[33] Wu J, Li L, Jiang G, Zhan H, Zhu X, Yang W. NCAPG2 facilitates glioblastoma cells' malignancy and xenograft tumor growth via HBO1 activation by phosphorylation. Cell Tissue Res. 2021; 383:693–706. 10.1007/s00441-020-03281-y 32897418

[34] Jing Z, Liu Q, Xie W, Wei Y, Liu J, Zhang Y, Zuo W, Lu S, Zhu Q, Liu P. NCAPD3 promotes prostate cancer progression by up-regulating EZH2 and MALAT1 through STAT3 and E2F1. Cell Signal. 2022; 92:110265. 10.1016/j.cellsig.2022.110265 35085770

[35] Schafer KA. The cell cycle: a review. Vet Pathol. 1998; 35:461–78. 10.1177/030098589803500601 9823588

[36] Suski JM, Braun M, Strmiska V, Sicinski P. Targeting cell-cycle machinery in cancer. Cancer Cell. 2021; 39:759–78. 10.1016/j.ccell.2021.03.010 33891890 PMC8206013

[37] Tang Z, Li C, Kang B, Gao G, Li C, Zhang Z. GEPIA: a web server for cancer and normal gene expression profiling and interactive analyses. Nucleic Acids Res. 2017; 45:W98–102. 10.1093/nar/gkx247 28407145 PMC5570223

[38] Li T, Fu J, Zeng Z, Cohen D, Li J, Chen Q, Li B, Liu XS. TIMER2.0 for analysis of tumor-infiltrating immune cells. Nucleic Acids Res. 2020; 48:W509–14. 10.1093/nar/gkaa407 32442275 PMC7319575

